# The relationship between stigma and alexithymia and their impact on satisfaction with care among people living with chronic obstructive pulmonary disease (COPD): A path analysis

**DOI:** 10.1371/journal.pone.0333599

**Published:** 2025-10-07

**Authors:** Keya Bibikar, Sanduni Madawala, Claire Hutton, Anne E. Holland, Chris Barton

**Affiliations:** 1 Department of General Practice, School of Public Health and Preventive Medicine, Monash University, Melbourne, Victoria, Australia; 2 Faculty of Pharmacy and Pharmaceutical Sciences, Centre for Medicine Use and Safety, Monash University, Melbourne, Victoria, Australia; 3 Respiratory Research @ Alfred, School of Translational Medicine, Monash University, Melbourne, Victoria, Australia; 4 Departments of Physiotherapy and Respiratory Medicine, Alfred Health, Melbourne, Victoria, Australia; Tehran University of Medical Sciences, IRAN, ISLAMIC REPUBLIC OF

## Abstract

**Introduction:**

Stigma is a common experience for individuals diagnosed with chronic obstructive pulmonary disease (COPD). It may elicit a set of emotional responses, consistent with alexithymia, that hinders conversations with healthcare providers. Alexithymia describes a set of cognitive-affective traits linked with difficulty identifying and distinguishing emotions. This study explored whether anticipated stigma in healthcare settings among patients with COPD is associated with alexithymia and whether it predicts satisfaction with care from healthcare providers.

**Methods:**

An online survey comprising validated questions was distributed between May and June 2024 to individuals who self-reported a doctor-diagnosis of COPD. Participants were recruited from advertisements on social media, online COPD support groups, and previous related studies. Correlation and multiple linear regression analyses were conducted to examine relationships between variables. Structural equation modelling (SEM) was used to explore these relationships through path analysis.

**Results:**

Respondents (n = 123) ranged in age from 33 to 90 years. Greater anticipated stigma was associated with higher levels of alexithymia (r = 0.278, p = 0.002) and lower satisfaction with care (r = −0.462, p < 0.001). In the path analysis, social support emerged as a mediating factor between alexithymia and anticipated stigma. As alexithymia increased, satisfaction with social support decreased, which was associated with higher anticipated stigma and reduced satisfaction with care from healthcare providers.

**Conclusion:**

This study helps clarify the association between alexithymia and anticipated stigma among people with COPD and how this impacts satisfaction with care. The findings signal a need to raise awareness of alexithymia and implement interventions to reduce stigma and enhance social support resources.

## Introduction

Chronic Obstructive Pulmonary Disease (COPD) is a progressive, life-limiting disease, characterised by persistent respiratory symptoms associated with chronic airflow obstruction; breathlessness, cough, and sputum [1]. These symptoms cause difficulty in performing daily activities such as walking, socialising, and exercising [2–4]. COPD is common in people with a history of cigarette smoking and together with this symptom profile people living with COPD experience stigma in a variety of settings, including health care settings [5,6].

When experienced in healthcare settings, stigma can pose a significant barrier to appropriate and effective care for individuals with chronic illness [7]. Healthcare providers may spend less time with, and offer less comprehensive education to patients with stigmatised conditions [8]. COPD patients report healthcare providers can be apathetic about their care once they become aware of their smoking history [6]. Anticipated stigma in healthcare settings has been associated with worse experience of care among people with COPD, leading to delayed or avoided visits to health care providers for management of COPD [9–11]. Understanding the impact and consequences of stigma in health care settings is an important issue for improving the quality of care for people living with chronic respiratory illness.

Recent literature suggests that stigma might trigger a set of emotional responses characteristic of the psychological trait alexithymia [12]. Alexithymia describes a set of emotional characteristics associated with difficulty identifying and describing emotions, identifying feelings, finding the words to describe them, and distinguishing emotions from body sensations [13–15]. The prevalence of alexithymia in the general population is estimated at approximately 13%, but is higher among men and individuals with a history of trauma [16,17]. Alexithymia has been linked to stigma in various conditions including epilepsy, inflammatory bowel disease, depression, psoriasis, and among burn survivors [12,18].

Alexithymic people can exhibit poorer emotion regulation and reduced capacity to manage negative emotions [19]. These characteristics may hinder open communication and shared decision-making with healthcare providers, impacting engagement in care, the doctor-patient relationship, and satisfaction with communication. Providers may misinterpret signs or symptoms of disease severity, resulting in delayed diagnosis, under treatment, or unsuitable medication regimens [20]. Such experiences may reinforce perceptions of stigma and judgement in healthcare settings, as reported by people with COPD [6,9].

Beyond individual emotional traits, factors such as social support may also influence how people with COPD experience stigma and engage with healthcare. Social support refers to the perceived availability and quality of emotional and practical assistance from family, friends, and the broader community [21]. It has been shown to buffer the psychological impact of chronic illness, support emotional regulation, and improve engagement with healthcare services [3,4]. Alexithymia is associated with lower quality interpersonal relationships, which tend to be shallow or superficial, limiting access to meaningful support [22]. These interpersonal deficits may also hinder the development of a therapeutic alliance and reduce satisfaction with care.

Taken together, individuals with COPD who exhibit traits of alexithymia may be more vulnerable to experiencing stigma in healthcare settings. This vulnerability may stem from difficulties in emotional expression and interpersonal communication, which can negatively impact the patient-provider relationship and satisfaction with care. This study aimed to explore the relationship between alexithymia and stigma in the context of COPD, a chronic and stigmatised illness. We hypothesised that individuals reporting a diagnosis of COPD who score higher on a scale of alexithymia will anticipate greater stigma in healthcare settings and report lower satisfaction with care.

## Methods

### Study design and setting

**Fig 1:**
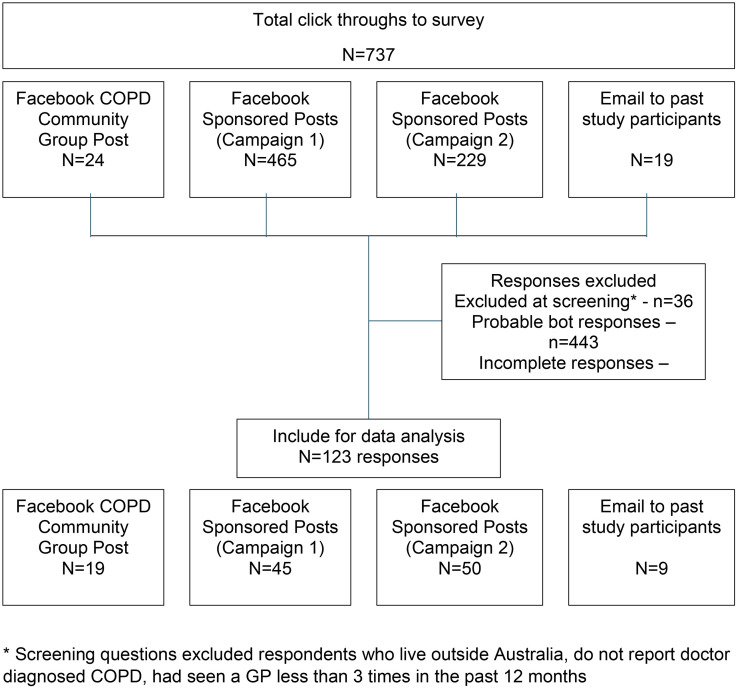
Flow diagram showing sources of participant recruitment and reasons respondents were excluded.

A cross-sectional survey was distributed via multiple channels using the Monash University licensed Qualtrics^TM^ platform. Participants were recruited through paid advertisements on Facebook (the most used social media platform in Australia), posts in online COPD community groups (also on Facebook), and from a list of participants in a previous study who consented to be recontacted by the research team [10] (Fig 1). Paid advertisements targeted users living in Australia aged thirty years and over. Separate advertisement tiles were created for men and women to tailor messaging and encourage participation.

Eligibility criteria included self-reported doctor-diagnosed COPD (“Has a doctor ever told you that you have Chronic Obstructive Pulmonary Disease (COPD), emphysema, or chronic bronchitis?”), age greater than 30 years, and at least three visits to a general practitioner (GP) in the past 2 years. Participants were required to be residing in Australia to ensure consistency in healthcare system exposure and sociocultural context.

A monetary incentive was offered in the form of a prize draw to win one of 10 AUD$50 e-gift cards, to increase response rate. Screening questions at the beginning of the survey were used to exclude individuals who did not meet the inclusion criteria. The survey was available only in English and had to be completed online. No other exclusion criteria were applied.

The study was designed as an exploratory analysis to investigate relationships between stigma, alexithymia, and satisfaction with care among individuals living with COPD. The sample size was informed by a previous online survey conducted by our group, which recruited 163 participants with COPD using a similar online strategy and identified statistically significant differences in anticipated stigma between chronic illness groups [10]. Based on this precedent, we aimed to recruit a comparable number of participants to ensure sufficient variability in key measures and enable multivariate analyses, including path analysis.

Ethics approval for the study was granted by the Monash University Human Research Ethics Committee (#40886). The survey landing page provided information and a link to a detailed explanatory statement. Participants indicated consent by clicking through to the online survey.

### Data collection

Valid and reliable scales were used to measure primary variables of interest including alexithymia, anticipated stigma and satisfaction with care. The online survey was pilot tested by a small number of consumer advisors with chronic illness for flow and estimate completion time (pilot data were excluded from analysis). The final survey was available via an online link between May and June 2024 and incorporated extensive protections against fraudulent responses (e.g., bots), including reCAPTCHA scoring and ‘honeypot’ questions designed to identify and exclude non-human responders. A copy of the survey is available in S1File.

#### Alexithymia.

Alexithymia was assessed using the Toronto Alexithymia Scale (TAS-20). The TAS-20 is considered the ‘gold standard’ measure of alexithymia [23,24]. Each item is scored from 1 to 5 across 20 questions, with scores summed to obtain a total score. Scores greater than 60 indicate high alexithymia, scores between 52–60 indicate borderline alexithymia and scores below 51 indicate low or no alexithymia [23,25]. Cronbach’s alpha was calculated as an indicator of internal reliability in our sample and was considered good (α = 0.85).

#### Anticipated stigma.

Anticipated stigma was measured using the Chronic Illness Anticipated Stigma Scale (CIASS) [26]. The 4-item healthcare settings subscale was used to assess the degree to which participants anticipated stigma and stigmatising behaviours in healthcare settings. Anticipated stigma (CIASS) scores ranged from 1 to 5 and were averaged for analysis, with higher scores indicating stronger feelings of anticipated stigma. Internal reliability in our sample was considered good (α = 0.88).

#### Communication and satisfaction with care.

Satisfaction with healthcare providers was measured using the 7-item Short Assessment of Patient Satisfaction (SAPS) scale [27]. This is a valid and reliable instrument developed to capture core dimensions of patient satisfaction, particularly those related to interpersonal processes such as communication, empathy, and trust. It was designed based on a conceptual model that integrates cognitive and affective components of satisfaction reflecting patients’ evaluations of care quality and their emotional responses to that care [27]. Scores range from 0 to 28, with higher scores indicating greater satisfaction. Internal reliability in our sample was acceptable (α = 0.76).

#### Sociodemographic and other variables.

Sociodemographic variables including age, gender, marital status, smoking history, and employment status, were assessed using items from previous Australian population surveys [28]. Satisfaction with social support was assessed using the seven item subjective support subscale of the Duke Social Support Index [29]. Items reflect how content individuals are with support received from family, friends, or others in their social network. Satisfaction with social support was categorised as low (score 7–11), medium (12–16) or high (17+).

Additional variables of interest including depression and anxiety (DASS10) [30], quality of life (EQ-5D) [31], self-efficacy (The single item self-efficacy question) [32], self-management capability (Patient Activation Measure) [33] and medication adherence [34] were assessed using validated scales. COPD severity was estimated using the modified Medical Research Council (mMRC) dyspnoea scale [35] Respondents’ postcodes were used to confirm Australian residency and to assign a score of relative socioeconomic advantage or disadvantage using the Socio-Economic Index for Areas (SEIFA) [36].

#### Data analysis.

Data from each recruitment channel were captured in standalone databases to minimise the risk of data corruption from fraudulent responses. Each dataset was carefully checked to identify and remove any responses potentially arising from bots including reCAPTCHA score <0.5 and visual inspection of meta-data such as time to complete, geolocation and IP address. Cleaned datasets were combined into a single database in IBM SPSS Version 29 for analysis. Imputation was not used where there was missing data. Participants with incomplete responses to key variables of interest—alexithymia, anticipated stigma, or satisfaction with care—were excluded from the final analysis. These exclusions are reflected in Fig 1 under ‘incomplete responses.’

Bivariate analyses were conducted using correlation, independent sample t-tests or one-way ANOVA to examine relationships between sociodemographic variables and the primary variables of interest (alexithymia, anticipated stigma and satisfaction with care).

A series of multivariate linear regression models were used to identify predictors of satisfaction, alexithymia and anticipated stigma (see S2 File). Variables with a p-value less than 0.2 in bivariate analyses, or with a sound theoretical justification, were considered for inclusion or exclusion in each multivariate regression model in line with standard practice for exploratory analyses. This threshold was applied uniformly across all bivariate analyses, including correlations, to identify candidate variables for inclusion in the path analysis. Independent predictors identified through regression were selected as potential candidates for inclusion in the path analysis [37] which aimed to confirm the direction of associations between alexithymia, anticipated stigma, and satisfaction, while accounting for factors such as social support. Gender was also included in the model given its relationship to alexithymia described in the literature previously [38].

Path analysis was conducted using structural equation modelling (SEM) in SPSS AMOS, Version 29. SEM is a confirmatory technique used as an extension to multiple regression, to illustrate direct and indirect relationships between variables through a path diagram [37,39]. The outputs also provide evidence of the strength of relationships and how the data fit the model [37]. The sample size was considered adequate for path analysis based on established guidelines suggesting the sample size should be at least 10 times the number of parameters [40].

## Results

Data from n = 123 participants was retained for analysis following careful screening and removal of responses that were indicative of fraudulent activity (i.e., bot-generated) (Fig 1). Participant characteristics are reported in Table 1. Ages ranged from 33 to 90 years (mean age 66.4 ± 10.1). Most respondents were female (n = 78, 63.4%) and most lived in disadvantaged socioeconomic areas. Participants reported moderate levels of satisfaction with social support, and just under half were married or in a domestic partnership (45.5%). The majority reported a history of smoking (n = 115, 93.5%), and approximately one-quarter continued to smoke (26.8%). Two-thirds (65.6%) reported mMRC grade 0–2, indicative of mild to moderate breathlessness. Nearly all reported having a regular General Practitioner (GP) (n = 118, 95.9%), and about half saw a specialist for management of COPD (n = 59, 48.0%).

**Table 1:** Differences in mean alexithymia, anticipated stigma, and satisfaction with care total scores by sociodemographic characteristics of participants.

Demographic variable	Category	n, (%)	TAS-20 Mean (St Dev)	CIASS Mean (St Dev)	SAPS Mean (St Dev)
All participants		N = 123	56.9 (11.2)	2.1 (0.8)	15.6 (2.8)
Age	<65 years	N = 47 (38.2%)	57.8 (10.7)	2.3 (0.9)	15.2 (2.9)
	65-74 years	N = 55 (44.7%)	57.4 (10.6)	2.1 (0.7)	15.7 (2.7)
	>75 years	N = 21 (17.1%)	52.5 (12.8)	1.8 (0.6)	16.3 (2.7)
Gender	Male	N = 41(33.3%)	61.4 (10.6)***	2.2 (0.8)	15.7 (2.6)
	Female	N = 78 (63.4%)	54.5 (10.8)	2.0 (0.7)	15.6 (2.9)
SEIFA quintile	Quintile 1	N = 24 (19.5%)	58.0 (11.7)	2.1 (0.8)	15.3 (3.2)
	Quintile 2	N = 24 (19.5%)	57.2 (9.3)	2.1 (0.8)	16.2 (2.1)
	Quintile 3	N = 24 (19.5%)	57.3 (12.3)	2.1 (0.8)	14.7 (2.8)
	Quintile 4	N = 24 (19.5%)	55.2 (11.7)	2.1 (0.8)	15.3 (2.8)
	Quintile 5	N = 27 (22%)	55.9 (11.1)	2.2 (0.7)	16.4 (2. 6)
Satisfaction with social support	Low (REF)	N= (10.9%)	66.5 (12.0)	2.8 (0.9)	14.5 (2.3)
	Medium	N= (64.7%)	57.6 (9.7)**	2.1 (0.7)**	15.5 (2.9)
	High	N= (24.4%)	50.2 (11.0)***	1.8 (0.6)***	16.3 (2.5)
Marital status	Single/never (REF)	N = 11 (8.9%)	58.6 (8.1)	2.6 (1.0)	13.4 (2.8)
	Married/Domestic partnership	N = 56 (45.5%)	55.9 (11.2)	2.0 (0.7)*	15.8 (2.7)*
	Divorced/widow/ separated	N = 56 (45.5%)	54.1 (12.1)	2.1 (0.7)	15.8 (2.5)*
Education	Did not complete Yr12	N = 50 (40.7%)	58.4 (9.6)	1.8 (0.7)	16.1 (2.9)
	Completed Yr12	N = 10 (8.1%)	56.2 (7.7)	1.8 (0.6)	14.7 (2.6)
	TAFE/diploma:	N = 44 (35.8%)	54.3 (11.9)	2.1 (0.8)	15.8 (2.5)
	3-year degree	N = 12 (9.8%)	49.2 (15.13)	2.3 (0.5)	14.8 (2.9)
	>3-year degree:	N = 9 (5.7%)	44.6 (12.46)	2.4 (1.0)	15.2 (2.2)
Employment status	Employed	N= (12.2%)	53.25 (12.1)	2.1 (0.9)	14.8 (3.6)
	Retired/ Pensioner	N= (74.0%)	54.69 (10.8)	1.9 (0.7)	15.9 (2.6)
	Unemployed	N= (13.8%)	61.13 (14.4)	2.0 (0.9)	15.3 (2.1)
Smoking habits	Current smoker (REF)	N = 33 (26.8%)	59.79 (10.82)	2.49 (0.8)	15.3 (3.0)
	Past smoker	N = 82 (66.7%)	55.29 (11.36)	1.96 (0.7)**	15.7 (2.7)
	Never smoked	N = 8 (6.5%)	58.75 (7.42)	2.03 (0.6)	15.6 (2.5)
Past smokers	Time Quit:				
	1-3 years ago	N = 16 (13%)	56.6 (12.0)	1.8 (0.7)	15.8 (3.3)
	3-5 years ago	N = 11 (8.9%)	57.4 (13.3)	2.3 (1.0)	16.1 (2.0)
	>5 years ago	N = 54 (43.9%)	54.3 (10.9)	1.9 (0.7)	15.7 (2.6)
E-Cigarette use	Current user	N = 9 (7.3%)	50.5 (12.0)	2.3 (0.4)	14.6 (2.6)
	Past user	N = 14 (11.4%)	55.6 (13.9)	2.2 (0.9)	15.9 (3.0)
	Never Used	N = 100 (81.3%)	55.3 (11.1)	2.1 (0.8)	15.7 (2.7)

TAS-20 – Toronto Alexithymia Scale, CIASS – Chronic Illness Anticipated Stigma Scale, SAPS – Short Assessment of Patient Satisfaction. TAFE = technical college, SEIFA = Socioeconomic Index For Area.

* Indicates differences between groups are statistically significant at 0.05 level (p < 0.05); ** 0.01 level (p < 0.01); *** 0.001 level (p < 0.001).

Mean and Standard Deviation (St Dev) scores for alexithymia, anticipated stigma, and satisfaction with care are presented in Table 1, along with differences across sociodemographic characteristics. In total, n = 54 (43.9%) participants had a TAS-20 score above 60, exhibiting high alexithymia traits; n = 32 (26%) had borderline alexithymia; and n = 37 (30.1%) had low or no alexithymia traits. Male respondents and those less satisfied with social support reported higher TAS-20 scores. Anticipated stigma was weakly correlated with younger age (r = −0.199, p = 0.027) although no significant differences were observed between age categories. Individuals who were less satisfied with social support, single/never married, and current smokers had higher anticipated stigma scores. Satisfaction with care was lowest amongst single/never married respondents but did not differ significantly across other sociodemographic characteristics.

Correlations between anticipated stigma, alexithymia, and patient satisfaction were explored in a correlation matrix (Table 2). Alexithymia was weakly and positively correlated with anticipated stigma (r = 0.278, p = 0.002), but not with satisfaction (r = −0.117, p = 0.196). Bivariate correlations between Depression Anxiety and Stress Scale total score, patient activation, self-efficacy, medication adherence, and quality of life are shown in Table 2.

**Table 2:** Correlation matrix.

	CIASS	TAS-20	SAPS
CIASS			
TAS-20	r = 0.278** **p = 0.002**		
SAPS	r = −0.462*** **p < 0.001**	r = −0.117 p = 0.196	
DASS-10	r = 0.251** **p = 0.005**	r = 0.656*** **p < 0.001**	r = −0.193* **p = 0.034**
PAM	r = −0.502*** **p < 0.001**	r = −0.306*** **p < 0.001**	r = 0.244** **p = 0.007**
Self-efficacy	r = −0.283** **p = 0.002**	r = −0.280** **p = 0.002**	r = 0.091 p = 0.319
Medication Adherence	r = −0.259** **p = 0.004**	r = −0.156 p = 0.085	r = 0.141 p = 0.120
EQ5D	r = −0.117 p = 0.199	r = −0.314*** **P < 0.001**	r = 0.195* **p = 0.031**
mMRC	r = 0.036 p = 0.697	r = 0.128 p = 0.160	r = −0.014 p = 0.880

CIASS – Chronic Illness Anticipated Stigma Scale, TAS-20 – Toronto Alexithymia Scale, SAPS – Short Assessment of Patient Satisfaction, DASS-10 – Depression Anxiety Stress Scale, PAM – Patient Activation Measure, GSE – Single item self-efficacy question, EQ-5D – EuroQuol-5D, mMRC - modified Medical Research Council scale.

*Indicates bivariate correlation is statistically significant at 0.05 level (p < 0.05); ** 0.01 level (p < 0.01); *** 0.001 level (p < 0.001)

Multivariate regression analyses are detailed in S2 File. Anticipated stigma was the only independent predictor of satisfaction scores (β = −0.43, p < 0.001), explaining 20.7% of the variance in SAPS score.

### Path analysis

**Fig 2:**
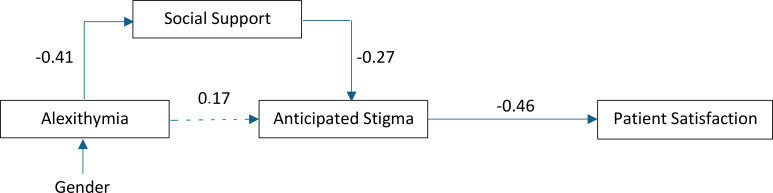
Path analysis diagram illustrating the associations between alexithymia, social support, anticipated stigma and patient satisfaction, with their respective correlation coefficients. Solid lines represent a significant correlation is present. Dotted lines represent non-statistically significant correlations.

Satisfaction with care was identified as the end point that optimised model fit (Fig 2). Social support was a mediating factor between alexithymia and anticipated stigma, which in turn preceded the path between anticipated stigma and satisfaction with care (β = −0.46, p < 0.001) (Fig 2). Gender was included in the model as a covariate due to its significant association with alexithymia. Squared multiple correlations indicated that the model explained 17% of the variance in social support, 13.8% in anticipated stigma and 21.4% in satisfaction with communication. These findings suggest that higher alexithymia contributes to lower satisfaction with social support, which is associated with greater anticipated stigma and, subsequently, reduced satisfaction with care.

Structural equation model indices demonstrated good model fit (χ^2^ = 1.457, df = 5, p = 0.918) with all predictor variables aligned with the hypothesised direction. The model demonstrated a CMIN/DF ratio of 0.291, and Comparative Fit Index (CFI) greater than 0.95 (CFI = 1.000). The RMSEA was 0.000 (90% CI [0.000, 0.045]), with a PCLOSE value of 0.956, and TLI 1.000 indicative of good fit.

Additional downstream consequences of reduced satisfaction with communication – such as self-management capability, medication adherence and quality of life – were explored. However, models predicting these outcomes were not statistically significant (data not shown).

## Discussion

This study examined the relationship between anticipated stigma in healthcare settings and alexithymia among people with COPD living independently in the community, and how these factors influenced satisfaction with healthcare providers. Anticipation of stigma was common in this cohort, and many participants also exhibited traits of alexithymia. Respondents who anticipated stigma in healthcare settings exhibited greater alexithymic characteristics and reported lower satisfaction with care. The role of social support was important in these relationships, playing a mediating role between alexithymia and stigma.

The prevalence of alexithymia in our sample (43.9%) was higher than previously reported in Chinese (23.6%) [41] and Greek samples (12%) [42], but consistent with findings by Han et al [43] (42%) who reported a prevalence of 42% among COPD patients, particularly males, compared to healthy controls. The higher prevalence of alexithymia among males has been shown by others to be moderated by social constructs impacting emotional responses [38] and consistent with our findings, participants who perceived lower levels of social support scored more highly on alexithymia measures [44]. We also considered a range of psychological and behavioural variables, including depression, anxiety, self-efficacy, and patient activation. As observed in previous studies, we found a strong correlation between depression and anxiety scores and alexithymia [45]. However, social support emerged as the most significant mediating factor in our path analysis, linking alexithymia to anticipated stigma and, ultimately, to satisfaction with care.

Existing literature suggests that alexithymic individuals may underestimate both the physical and emotional components of illness, regardless of disease severity [20]. They may have difficulty expressing their emotions and symptoms to healthcare providers, which can negatively impact the doctor-patient relationship, particularly in terms of communication and trust [18]. For example, in early-stage COPD, a patient may experience fatigue, a common but non-specific symptom, and feel concerned enough to seek medical attention. However, a patient with alexithymia may struggle to articulate the extent of their concern or the emotional distress associated with the symptom, leading the clinician to misinterpret the presentation as less serious or attribute it to a benign cause, potentially delaying further investigation. More broadly, when patients with alexithymia present with somatic symptoms, that reflect emotional distress, the absence of clearly expressed emotional concern may cause doctors to underestimate the severity of the presentation or overlook the possibility of an underlying biomedical condition with a physiological cause. This can contribute to delayed diagnosis or under-treatment. Consequently, clinicians may find it challenging to assess the patient’s overall well-being and address their healthcare needs effectively and with empathy, as reported in qualitative research with COPD patients [9].

Effective communication is essential for accurate symptom reporting, reduced uncertainty, greater engagement in decision-making, appropriate utilization of health care services, improved adherence to treatment regimens, and better clinical outcomes [46]. Greater satisfaction with communication can foster patient confidence, encourage follow-up, and engagement in consultations, contributing to a more positive care experience. Failing to account for patient perceptions or fears of stigma in health care settings may contribute to dissatisfaction with communication and trust, as reported by individuals seeking care for COPD [5,6]. Although satisfaction with care in this study was assessed using the SAPS scale, its emphasis on interpersonal processes such as empathy and trust allows these findings to be interpreted as reflective of communication quality.

Zhang et al [12] hypothesised that stigma may “trigger” alexithymia, with the relationship influenced by an individual’s level of “resourcefulness”. Lower resourcefulness reflects reduced adaptability and coping capacity in the face of adversity [47]. In our study, however, the relationship between alexithymia and anticipated stigma was not direct but mediated by social support. Using path analysis, we found that the direction of association flowed from alexithymia to stigma, contrary to that proposed by Zhang et al. in their study of burn survivors [12]. In that context, visible changes in appearance were thought to increase fear of rejection and emotional suppression, potentially triggering alexithymia. While this directionality may be plausible in trauma-related contexts, our study focused on COPD, where alexithymia is more likely to represent a stable emotional processing trait. This trait may shape how individuals perceive and respond to social interactions, including those involving stigma. The gradual progression of COPD symptoms over time may also contribute to this difference in directionality. Longitudinal studies are needed to further explore the temporal and causal dynamics between alexithymia and stigma in populations with chronic illness.

### Strengths & limitations

This cross-sectional, survey-based study had several strengths. Recruitment of a nationwide sample of 123 participants with doctor-diagnosed COPD was a significant achievement, particularly given the challenges of engaging vulnerable populations with stigmatised chronic illness in research. Consistent with previous studies, we found that social media was a cost-effective way and efficient method for reaching a broad cross-section of individuals living with COPD across Australia [8]. We implemented extensive bot protections to protect against fraudulent responses to our online survey, and used validated and reliable scales to assess key outcomes. The path analysis demonstrated good model fit across most indices. The Tucker-Lewis Index (TLI) exceeded the typical upper bound of 1.000, which, while mathematically possible, is not interpretable beyond this threshold. This may reflect exceptionally good fit but more likely results from the simplicity of the model and the modest sample size. TLI values are typically capped at 1.000 and interpreted as indicating excellent model fit.

A key limitation of the study was the reliance on a convenience sample of community-dwelling individuals who self-reported a doctor-diagnosis of COPD. Self-report has been shown to be an acceptable proxy for medical record confirmation of COPD [48], recruitment was conducted exclusively online. As such, the sample may not be representative of the broader COPD population, limiting the generalisability of findings. Additionally, the survey was only available in English language and required internet access, which may have excluded some potential participants. This was a sample experiencing predominately mild to moderate disease overall, with more women than men, and most lived in socioeconomically disadvantaged areas. Moreover, due to the cross-sectional study design, only associations could be established, rather than causality.

Efforts were made to minimise respondent burden in the selection of instruments; however, the inclusion of multiple covariates necessitated a lengthy questionnaire, which may have contributed to respondent fatigue and influenced data quality. Further research utilising a longitudinal design, representative sampling and confirmation of lung disease by spirometry are needed to determine causality between alexithymia and stigma, and confirm or refute if alexithymia can be triggered by experiences of anticipated stigma in people living with COPD.

Further, people living with COPD are not alone in experiencing stigma in healthcare settings. Individuals with other medical conditions like chronic pain, mental illness, those who are incarcerated or who use substances, refugees or immigrants, and even patients with poorly controlled diabetes or obesity may also face stigma in healthcare. Confirming our findings in these broader populations would be valuable [49].

## Conclusions

This is the first study to establish a relationship between alexithymia and anticipated stigma among people with COPD and to examine its impact on satisfaction with healthcare. The findings highlight the important mediating role of social support in the relationship between alexithymia and anticipated stigma. The results have implications for clinical practice and identify several avenues for further research.

Our findings underscore the need to raise awareness of alexithymia and develop interventions that address the impacts of anticipated stigma in healthcare settings. Addressing stigma is crucial due to the strong impacts it has on communication with health care providers and experience of care. Promoting greater empathy and awareness may help reduce stigma in healthcare settings and foster more positive patient-provider interactions [9,25]. This is likely to improve patient outcomes and enhance both the care experience and satisfaction with care.

**AI use disclosure**

Artificial intelligence (AI) was used to assist with language editing and proofreading of this manuscript. Specifically, a generative AI tool (Microsoft CoPilot 365) was employed to improve grammar, clarity, sentence structure, and alignment with scientific writing conventions. All content was critically reviewed and verified by the authors to ensure accuracy and integrity. No AI-generated content was used without human oversight, and the authors take full responsibility for the final version of the manuscript.

## Supporting information

S1 File Study survey. (PDF)

S2 File Regression models to identify predictors of alexithymia, anticipated stigma, and satisfaction with care. (DOCX)
