# Supplementary material for: The relationship between stigma and alexithymia and their impact on satisfaction with care among people living with chronic obstructive pulmonary disease (COPD): A path analysis
Source: PLoS One. 2025 Oct 7;20(10):e0333599. doi: 10.1371/journal.pone.0333599 (PMC12503303; doi:10.1371/journal.pone.0333599)
Supplement: S1 File — (PDF) [file pone.0333599.s001.pdf]

Supporting Information 1 - Study Survey

Start of survey

Talking with health care providers about COPD.

Thank you for your interest in this research. This study is being conducted by researchers at Monash University and is hoping to understand more about how people with Chronic Obstructive Pulmonary Disease (COPD) interact with healthcare providers.

Detailed information about the study can be accessed through the link available **here**.

This survey will involve:

- Eligibility questions
- Questions about your experience with your GP
- How you feel about your care and any factors that impact communication
- Approx. 15 minutes to complete (you can take a break and come back within 1 week)
- Upon completion, you can enter a prize draw for the chance to win 1 of 10 $50 e-gift cards.

Your responses will be stored securely by Monash University and not shared with your healthcare providers.

By clicking next, you agree to participate.

Eligibility - 6 items

The following questions will determine your eligibility for this study. Please answer as accurately as possible.

Please indicate

☐ I'm not a robot
reCAPTCHA
Privacy - Terms

Please type your age (in years) below?

Do you currently live in Australia?

- ○ Yes
- ○ No

What is your postcode?

Has a doctor ever told you that you have Chronic Obstructive Pulmonary Disease(COPD), emphysema, or chronic bronchitis?

- ○ Yes
- ○ No

How many times have you seen a GP for your own health in the last 2 years?

- ○ 0 times
- ○ 1-2 times
- ○ 3- 4 times

- 5 or more times

How often do you now smoke cigarettes, pipes or other tobacco products?

- Daily
- At least once a week
- Less than weekly
- Not at all but I have been a regular smoker in the past
- Not at all and I have never been a regular smoker

Do you currently use e-cigarettes or vapes?

- Daily
- At least once a week
- Less than weekly
- Not at all but I have been a regular e-cigarette user in the past
- Not at all and I have never been an e-cigarette user

Bot detection

Your responses have been flagged as a potential bot. Please answer these two questions to confirm you are human.

Please indicate whether you are a bot (B) or a human (H), by typing the letter 'B' or 'H'?

What year is it?

For bots only

What is 3+2?

- 7
- 4
- 5

Non-eligibility message

Thank you for your interest in this survey, unfortunately you are not eligible for this study.
If you have any questions or concerns, please give us a call on 03 9903 0481.

Quitting - 2 items

When did you quit smoking cigarettes daily?

- In the last 6 months
- In the last 6 to 12 months
- In the last 1 to 3 years
- In the last 3 to 5 years
- More than 5 years ago

mMRC Dyspnoea Scale - 1 item

Can you describe your level of breathlessness?

- I only get breathless with strenuous exercise
- I get short of breath when hurrying on level ground or walking up a slight hill
- On level ground, I walk slower than people of the same age because of breathlessness, or have to stop for breath when walking at my own pace

- I stop for breath after walking about 100 yards or after a few minutes on level ground
- I am too breathless to leave the house or I am breathless when dressing

GP care - 13 items

The following questions in this survey will refer to all the General Practitioners (GPs) you visited in the past 12 months for your own health. Please think of these GPs you visited when answering the following questions.

Do you have a regular GP?

- Yes
- No

Were any of these visits for urgent medical care?

- Yes
- No

In the last 12 months, when you contacted the GP's office to get an appointment for urgent medical care, how often did you get an appointment as soon as you needed?

- Never
- Sometimes
- Usually
- Always

Did you see a GP for non-urgent medical care?

- Yes
- No

In the last 12 months, when you made an appointment for non-urgent medical care, how often did you get an appointment as soon as you needed?

- Never
- Sometimes
- Usually
- Always

In the last 12 months has there been any time you needed to see a GP but delayed going?

- Yes
- No

In the last 12 months, has there been any time you needed to go to a GP but didn't?

- Yes
- No

Using any number from 0 to 10, where 0 is the worst care possible and 10 is the best care possible, what number would you use to rate the care you received from GPs?

0 1 2 3 4 5 6 7 8 9 10

Using any number from 0-10, where 0 means that you don't trust this GP and 10 means you trust this GP completely, what number would you use to rate how much you trust this doctor?

0 1 2 3 4 5 6 7 8 9 10

Has a GP ever referred you to a pulmonary rehabilitation program?

- Yes
- No

Short Assessment of Patient Satisfaction - 7 items

The following questions will ask you to reflect on your experience communicating with your GP.

How satisfied are you with the effect of your treatment/care?

- Very Satisfied
- Satisfied
- Neither satisfied nor dissatisfied
- Dissatisfied
- Very dissatisfied

How satisfied are you with the explanations the doctor/other health professional has given you about the results of your treatment/care?

- Very Satisfied
- Satisfied
- Neither satisfied nor dissatisfied
- Dissatisfied
- Very dissatisfied

The doctor/other health professional was very careful to check everything when examining you.

- Strongly agree
- Agree
- Not sure
- Disagree
- Strongly disagree

How satisfied were you with the choices you had in decisions affecting your health care?

- Very Satisfied
- Satisfied
- Neither satisfied nor dissatisfied
- Dissatisfied
- Very dissatisfied

How much of the time did you feel respected by the doctor/other health professional?

- All of the time
- Most of the time
- About half the time
- Some of the time
- None of the time

The time you had with the doctor/other health professional was too short.

- Strongly agree
- Agree
- Not sure
- Disagree
- Strongly disagree

Are you satisfied with the care you received in the clinic?

- Very Satisfied
- Satisfied
- Neither satisfied nor dissatisfied
- Dissatisfied
- Very dissatisfied

Chronic Illness Anticipated Stigma Scale - 4 items

The following questions will ask you to think about how healthcare workers such as doctors will treat you in the future. How likely is it that they will treat you in the following ways because of your chronic illness?

A healthcare worker will be frustrated with you.

- ◯ Very unlikely
- ◯ Unlikely
- ◯ Somewhat likely
- ◯ Likely
- ◯ Very likely

A healthcare worker will give you poor care.

- ◯ Very unlikely
- ◯ Unlikely
- ◯ Somewhat likely
- ◯ Likely
- ◯ Very likely

A healthcare worker will blame you for not getting better.

- ◯ Very unlikely
- ◯ Unlikely
- ◯ Somewhat likely
- ◯ Likely
- ◯ Very likely

A healthcare worker will think that you are a bad patient.

- Very unlikely
- Unlikely
- Somewhat likely
- Likely
- Very likely

Toronto Alexithymia Scale (TAS-20) - 20 items

Please read the following questions carefully and respond to indicate how you feel. The questions measure how you identify and describe emotions and symptoms.

	Strongly Disagree	Disagree	Neither agree nor disagree	Agree	Strongly agree
I am often confused about what emotion I am feeling.	○	○	○	○	○
It is difficult for me to find the right words for my feelings.	○	○	○	○	○
I have physical sensations that even doctors don't understand.	○	○	○	○	○
I am able to describe my feelings easily.	○	○	○	○	○
I prefer to analyze problems rather than just describe them.	○	○	○	○	○
When I am upset, I don't know if I am sad, frightened, or angry.	○	○	○	○	○
I am often puzzled by sensations in my body.	○	○	○	○	○
I prefer to just let things happen rather than to understand why they turned out that way.	○	○	○	○	○
I have feelings that I can't quite identify.	○	○	○	○	○

	Strongly Disagree	Disagree	Neither agree nor disagree	Agree	Strongly agree
Being in touch with emotions is essential.	○	○	○	○	○
I find it hard to describe how I feel about people.	○	○	○	○	○
People tell me to describe my feelings more.	○	○	○	○	○
I don't know what's going on inside me.	○	○	○	○	○
I often don't know why I am angry.	○	○	○	○	○
I prefer talking to people about their daily activities rather than their feelings.	○	○	○	○	○
I prefer to watch "light" entertainment shows rather than psychological dramas.	○	○	○	○	○
It is difficult for me to reveal my innermost feelings, even to close friends.	○	○	○	○	○
I can feel close to someone, even in moments of silence.	○	○	○	○	○
I find examination of my feelings useful in solving personal problems.	○	○	○	○	○
Looking for hidden meanings in movies or plays distracts from my enjoyment.	○	○	○	○	○

Half-way point

You are half-way through the survey, please keep going.

Patient Activation Measure - 13 items

The following questions ask you about the self-management of your health condition.

When all is said and done, I am the person who is responsible for managing my health condition.

- Strongly disagree
- Disagree
- Agree
- Strongly agree

Taking an active role in my own health care is the most important factor in determining my health and ability to function.

- Strongly disagree
- Disagree
- Agree
- Strongly agree

I am confident that I can take actions that will help prevent or minimize some symptoms or problems associated with my health condition.

- Strongly disagree
- Disagree
- Agree
- Strongly agree

I know what each of my prescribed medications do.

- Strongly disagree
- Disagree

- Agree
- Strongly agree

I am confident that I can tell when I need to go get medical care and when I can handle a health problem myself.

- Strongly disagree
- Disagree
- Agree
- Strongly agree

I am confident I can tell my health care provider concerns I have even when he or she does not ask.

- Strongly disagree
- Disagree
- Agree
- Strongly agree

I am confident that I can follow through on medical treatments I need to do at home.

- Strongly disagree
- Disagree
- Agree
- Strongly agree

I understand the nature and causes of my health condition.

- Strongly disagree
- Disagree
- Agree
- Strongly agree

I know the different medical treatment options available for my health condition.

- Strongly disagree
- Disagree
- Agree
- Strongly agree

I have been able to maintain the lifestyle changes for my health that I have made.

- Strongly disagree
- Disagree
- Agree
- Strongly agree

I know how to prevent further problems with my health condition.

- Strongly disagree
- Disagree
- Agree
- Strongly agree

I am confident I can figure out solutions when new situations or problems arise with my health condition.

- Strongly disagree
- Disagree
- Agree
- Strongly agree

I am confident that I can maintain lifestyle changes like diet and exercise even during times of stress.

- Strongly disagree

- Disagree
- Agree
- Strongly agree

Generalised Self-Efficacy Scale - 1 item

I am confident in my ability to solve problems that I might face in life (For example: I can usually handle whatever comes my way, If I try hard enough I can overcome difficult problems, I can stick to my aims and accomplish my goals).

Disagree strongly — Agree strongly

0 1 2 3 4 5 6 7 8 9 10

Medication Adherence - 4 items

The following questions will ask you about your medication habits and behaviours. Please answer as accurately as possible.

Do you ever forget to take your medicine?

- Yes
- No

Are you careless at times about taking your medicine?

- Yes
- No

When you feel better do you sometimes stop taking your medicine?

- Yes
- No

Sometimes if you feel worse when you take the medicine, do you stop taking it?

- Yes
- No

Depression Anxiety Stress Scale (DASS) - 10-items

Please read each statement and choose from the options that indicates how much the statement applied to you over the past week. There are no right or wrong answers. Do not spend too much time on any statement.

	Never	Sometimes	Often	Almost Always
I felt I was close to panic				
I found it difficult to work up the initiative to do things				
I felt down hearted and blue				
I was intolerant of anything that kept me from getting on with what I was doing				
I felt that I had nothing to look forward to				
I felt scared without any good reason				
I tended to over-react to situations				
I was worried about situations in which I might panic and make a fool of myself				
I found it difficult to relax				
I couldn’t seem to experience any positive feelings at all				

If these questions have raised any concerns about your wellbeing, please contact your health care professional.

EQ-5D - 7 items

Under each heading, please tick the ONE box that best describes your health TODAY

Mobility

- I have no problems in walking about
- I have slight problems in walking about
- I have moderate problems in walking about
- I have severe problems in walking about
- I am unable to walk about

Self-care

- I have no problems washing or dressing myself
- I have slight problems washing or dressing myself
- I have moderate problems washing or dressing myself
- I have severe problems washing or dressing myself
- I am unable to wash or dress myself

Usual activities

- I have no problems doing my usual activities
- I have slight problems doing my usual activities
- I have moderate problems doing my usual activities
- I have severe problems doing my usual activities
- I am unable to do my usual activities

Pain/Discomfort

- I have no pain or discomfort
- I have slight pain or discomfort
- I have moderate pain or discomfort
- I have severe pain or discomfort
- I have extreme pain or discomfort

Anxiety/Depression

- I am not anxious or depressed
- I am slightly anxious or depressed
- I am moderately anxious or depressed
- I am severely anxious or depressed
- I am extremely anxious or depressed

We would like to know how good or bad your health is TODAY. This scale is numbered from 0 to 100. 100 means the best health you can imagine. 0 means the worst health you can imagine. Please drag the slider to indicate how your health is TODAY.

The worst health you can imagine						The best health you can imagine				
0	10	20	30	40	50	60	70	80	90	100

Now, write the number you marked on the scale in the box below. Your health TODAY is =

Sociodemographic information - 7 items

The following questions will ask you about your sociodemographic information. Please answer as accurately as possible.

In the last 12 months, how many times did you see a GP for your own health?

Other than COPD, emphysema or chronic bronchitis, has a doctor told you whether you currently have any of the following health conditions? (tick all that apply)

- ☐ Hypertension (high blood pressure)
- ☐ Heart Disease
- ☐ Stroke or stroke related loss of function
- ☐ Diabetes
- ☐ Asthma
- ☐ Lung Cancer
- ☐ Depression
- ☐ Anxiety
- ☐ None of these
- ☐ Do you have any other long term illnesses? If so, please specify below

Do you see a specialist for the management of your COPD?

- ○ Yes
- ○ No

Do you see a specialist for the management of any other chronic conditions you may have?

- Yes
- No
- Not applicable

What is your marital status

- Single, never married
- Married or domestic partnership
- Widowed
- Divorced
- Separated

What is your current primary employment status?

- Full-time, part-time or casual paid work
- Self-employed
- Retired/ Pensioner
- Disability support pension
- Doing unpaid work / volunteering
- Studying
- Looking after home/family
- Unemployed
- Other

What is your gender?

- Male
- Female
- Non-binary / gender diverse

- My gender isn't listed. I identify as ____
- Prefer not to say

What is the highest grade or level of school that you have completed?

- Some high school, but did not complete Year 12
- Completed Year 12
- TAFE/ diploma graduate
- Completed a 3-year University degree
- Completed more than 3-year University degree

Satisfaction with social support sub-scale - 7 items

The following questions will ask you about your interactions with your family and friends.

Please read each statement carefully when answering

	Hardly ever	Some of the time	Most of the time
Does it seem that your family and friends (people who are important to you) understand you?	○	○	○
Do you feel useful to your family and friends (people important to you)?	○	○	○
Do you know what is going on with your family and friends?	○	○	○
When you are talking with your family and friends, do you feel you are being listened to?	○	○	○

	Hardly ever	Some of the time	Most of the time
Do you feel you have a definite role (place) in your family and among your friends?	○	○	○
Can you talk about your deepest problems with at least some of your family and friends?	○	○	○

How satisfied are you with the kinds of relationships you have with your family and friends?

- ○ Very dissatisfied
- ○ Somewhat dissatisfied
- ○ Satisfied

End Block

That's the end of the survey, thank you for your participation. If you would like to receive a summary of the study results, please enter your email address by clicking on the following link. Your contact details will be kept separate from your survey responses to protect your anonymity.

We are also hoping you can help us recruit additional people who may have interest in completing the survey,

Please follow this link to enter your details

Your responses have been saved, you may now close the survey.

Powered by Qualtrics
