# Supplementary material for: The relationship between stigma and alexithymia and their impact on satisfaction with care among people living with chronic obstructive pulmonary disease (COPD): A path analysis
Source: PLoS One. 2025 Oct 7;20(10):e0333599. doi: 10.1371/journal.pone.0333599 (PMC12503303; doi:10.1371/journal.pone.0333599)
Supplement: S2 File — (DOCX) [file pone.0333599.s002.docx]

**Supporting Information 2: Regression models to identify predictors of Alexithymia, Anticipated Stigma, and Satisfaction with care.**

For each regression model variables from univariate analyses with p value <0.2, or variables with a strong theoretical reason for inclusion were included in the model.

**Model 1: Predictors of SAPS (total score) (Patient Satisfaction)**

The model was statistically significant (F = 4.525, df = 9, p<0.001 explaining 21.5% of the variance in SAPS total score by the independent variables in the model.

|  | Beta | T-statistic | Sig. | 95% CI for B |
| --- | --- | --- | --- | --- |
| **Anticipated stigma (CIASS)** | **-0.428** | **-4.729** | **<0.001**** | **-2.178 to -0.891** |
| Regular GP y/n | -0.128 | -1.506 | 0.135 | -4.049 to 0.552 |
| Marital status  (3 groups) | 0.122 | 1.338 | 0.184 | -0.250 to 1.291 |
| TAS 20 (total score) | 0.124 | 1.079 | 0.283 | -0.026 to 0.087 |
| EQ5D | 0.092 | 0.835 | 0.406 | -0.093 to 0.227 |
| Age^#^ | 0.046 | 0.510 | 0.611 | -0.037 to 0.063 |
| SEIFA Quintile | 0.054 | 0.639 | 0.524 | -0.220 to 0.429 |
| Social Support total score | 0.055 | 0.554 | 0.581 | -0.098 to 0.174 |
| DASS10 (total score) | -0.050 | -0.359 | 0.720 | -0.133 to 0.092 |

^#^ Variable included despite not meeting p<0.2 threshold in univariate analysis

Nb. Patient Activation Measure (PAM) excluded due to poor performance in the model.

**Model 2: Predictors of CIASS (anticipated stigma) average score**

The model was statistically significant (F = 4.525, df=10, p<0.001) explaining 37.2% of the variance in CIASS total score by the independent variables in the model.

|  | Beta | T-statistic | Sig. | 95% CI for B |
| --- | --- | --- | --- | --- |
| **Patient Satisfaction (SAPS total score)** | **-0.330** | **-4.093** | **<0.001** | **-0.137 to -0.048** |
| **Patient Activation Measure (total score)** | **-0.359** | **-3.599** | **<0.001** | **-0.054 to -0.016** |
| Smoking (current/past/never) | -0.165 | -1.944 | 0.055 | -0.476 to 0.005 |
| Social support | -0.052 | -0.550 | 0.584 | -0.046 to 0.026 |
| Marital status  (3 groups) | 0.035 | 0.421 | 0.674 | -0.156 to 0.239 |
| DASS10 (total score) | 0.065 | 0.513 | 0.609 | -0.022 to 0.037 |
| TAS-20 (Alexithymia) | 0.048 | 0.453 | 0.651 | -0.011 to 0.018 |
| EQ5D (quality of life) | -0.030 | -0.289 | 0.773 | -0.049 to 0.037 |
| Age | -0.022 | -0.262 | 0.793 | -0.015 to 0.011 |
| Self-Efficacy | 0.011 | 0.116 | 0.908 | -0.071 to 0.079 |

^#^ Variable included despite not meeting p<0.2 threshold in univariate analysis

**Model 3. Predictors of TAS-20 (alexithymia) total score**

The model was statistically significant (F = 10.571, df=11, p<0.001) explaining 49.4% of the variance in TAS-20 total score by the independent variables in the model.

|  | Beta | T-statistic | Sig. | 95% CI for B |
| --- | --- | --- | --- | --- |
| **DASS10** | **0.610** | **6.250** | **<0.001** | **0.708 to 1.368** |
| **Patient Activation** | **-0.240** | **-2.514** | **0.014** | **-0.636 to -0.075** |
| **Gender** | **-0.176** | **-2.485** | **0.015** | **-7.632 to -0.854** |
| Social support | -0.133 | -1.580 | 0.117 | -0.855 to 0.097 |
| EQ5D (QoL) | 0.134 | 1.237 | 0.219 | -0.245 to 1.054 |
| Age | -0.40 | -0.549 | 0.585 | -0.207 to 0.117 |
| Patient Satisfaction (SAPS total score) | 0.038 | 0.468 | 0.641 | -0.498 to 0.805 |
| Smoking habits | 0.032 | 0.379 | 0.705 | -2.765 to 4.070 |
| Self-Efficacy | 0.025 | 0.289 | 0.774 | -0.913 to 1.224 |
| Anticipated stigma (CIASS avg score) | -0.027 | -0.280 | 0.780 | -3.326 to 2.505 |
| Breathlessness (mMRC score) | -0.005 | -0.056 | 0.956 | -1.725 to 1.631 |

^#^ Variable included despite not meeting p<0.2 threshold in univariate analysis
